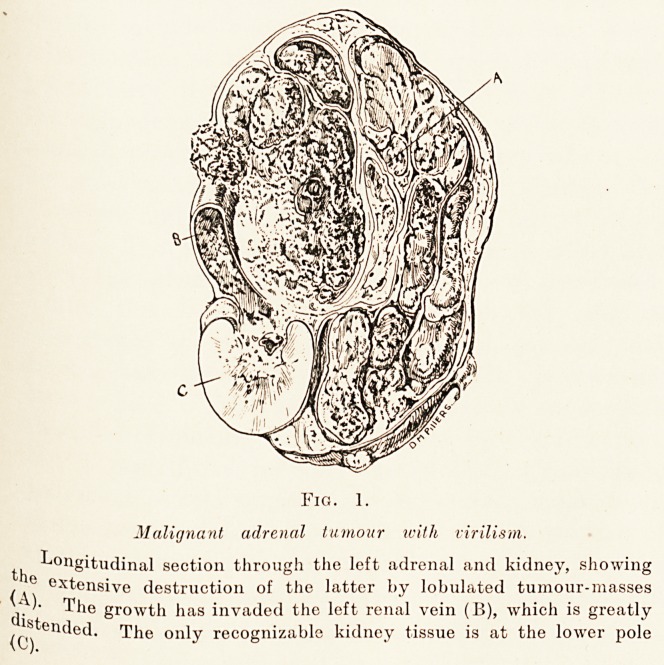# Suprarenal Virilism

**Published:** 1932

**Authors:** J. Antony Birrell

**Affiliations:** Physician, Children's Hospital, and Assistant Physician, General Hospital, Bristol


### SUPRARENAL VIRILISM.

BY

J. Antony Birrell, M.D., M.R.C.P.,

*Physician, Children's Hospital, and Assistant Physician, General Hospital, Bristol.*

---

The case, notes of which follow, is deemed worthy of record because the tumour found post-mortem offered an adequate explanation of the symptoms observed during life. The patient was shown recently at a meeting of the Bristol Medico-Chirurgical Society by the writer of this note.

Until a year ago the patient, a girl aged 5 years, was to all appearances normal, mentally and physically. During the last twelve months she became fat, listless, unusually silent, and prone to sit about, disinclined to take an interest in her surroundings or to play with other children.

With the increasing fatness growth in stature was at an abnormally vigorous rate, hair developing on the limbs and body generally. There was no menstruation.

Some few days before she was brought to my notice there had been attacks of shortness of breath which had caused alarm, and the indifference to her surroundings had become extreme.

When first seen by me she was ten pounds above the weight and six inches above the height of a girl of like age. She was very fat; her size was at once a striking feature. The fatness was most pronounced on the face, which was bloated and greasy, presenting a marked acne with comedones in all stages of maturity. There was a thick layer of fat over the abdomen (circumference 26 inches), clavicles and chest. She was prevailed upon with difficulty to stand, preferring to lie on her back in bed, speechless and motionless, quietly observing one's movements about the room. There was a coarse, dark and fully-developed pubic hirsuties, of female distribution, and fine hairs were obvious all over the trunk, upper arms and thighs, those on the back being about half an inch long.

The tongue was coated and constipation was marked. The heart-rate was 130, the sounds weak, and the blood-pressure 135/80—distinctly high for one of her age.

The nipples and surrounding areolæ were rather pronounced; there was, nevertheless, no actual breast development. The labia majora were unusually large and the clitoris markedly hypertrophic.

Blood urea, urea concentration and blood sugar tests revealed no abnormality, and a radiogram of the pituitary fossa was unexceptionable. Apart from the appearances outlined above clinical examination of the abdomen was negative.

After the lapse of a month the lower edge of the liver and the tip of the spleen became just palpable; the edge of the former now descended yet farther, almost down to the umbilicus, but no irregularity in its contour was obvious, and jaundice did not supervene. A resistance was now palpable deep in the right loin. The superficial mammary and epigastric venous anastomoses became very prominent. A fortnight after this enlargement of the liver was first noted, she complained of acute pain over its anterior surface, which was tender on pressure, and a fortnight later she died.

While she was under my observation the tongue was consistently moist, but furred; there was obstinate constipation and a quick pulse (130–150), with the mental and physical apathy referred to above.

### Note by Dr. A. L. Taylor.

*Post-mortem* a large tumour was found invading and largely destroying the left kidney, only the lower pole of which remained. Fig. 1 shows the tumour in longitudinal section. The growth was soft and lobulated and of a slaty colour, with large areas of necrosis and some hæmorrhage, but showing practically none of the lipoid characteristic of the normal adrenal cortex. Nevertheless, the absence of any recognizable adrenal gland on this side, together with the clinical features of the case, made it clear that the tumour was of adrenal and not of renal origin. The growth had extensively invaded the renal pelvis and the renal vein, which is seen greatly distended on the left of the illustration. In spite of this, the only discovered site of secondary growth was the liver, which contained several large round masses of soft hæmorrhagic appearance. The right suprarenal and right kidney were quite normal.

*Microscopically* the tumour has lost all resemblance to adrenal tissue, and consists of irregular strands and masses of atypical cells of varying size and shape, and of intensely malignant aspect. Numerous malignant giant cells and multinucleate cells are

FIG. 1.

*Malignant adrenal tumour with virilism.*

Longitudinal section through the left adrenal and kidney, showing the extensive destruction of the latter by lobulated tumour-masses (A). The growth has invaded the left renal vein (B), which is greatly distended. The only recognizable kidney tissue is at the lower pole (C).

present and mitotic figures abound. Most of the tumour is necrotic. In the liver-secondaries the epithelial origin of the tumour is quite unrecognizable. The cells are of unequal size, but for the most part of rounded or polygonal shape, with very densely staining nuclei and relatively scanty cytoplasm. The cells are loosely arranged in an indefinite rather fibrillary stroma richly supplied with ill-formed blood-vessels. Here also extensive areas of necrosis and hæmorrhage are present. Both primary and secondary growths are almost entirely free from lipoid material.

## Figures and Tables

**Fig. 1. f1:**